# 基于敞开式直接电离质谱技术的尿液中毒品检测

**DOI:** 10.3724/SP.J.1123.2022.01013

**Published:** 2022-07-08

**Authors:** Shiling XIONG, Huanhuan HONG, Luhong WEN, Shundi HU, Anqi CHEN, Wei XIONG, La CHEN

**Affiliations:** 1.宁波大学高等技术研究院, 浙江 宁波 315211; 1. Research Institute of Advanced Technologies, Ningbo University, Ningbo 315211, China; 2.宁波华仪宁创智能科技有限公司, 浙江 宁波 315100; 2. China Innovation Instrument Co., Ltd., Ningbo 315100, China

**Keywords:** 敞开式直接电离, 便携式质谱仪, 毒品, 尿液, 快速检测, ambient direct ionization, portable mass spectrometer, drug, urine, rapid detection

## Abstract

尿液作为一种易于获取的体内毒品检材,在吸毒人员快速筛查中被广泛应用。针对传统快速筛查技术存在假阳性率高、定量能力不足以及实验室质谱技术在快速检测中存在前处理复杂、检测耗时长、使用环境苛刻等问题,该文提出了一种基于敞开式直接电离质谱技术的生物样本快速检测方法。该研究采用探针式电喷雾离子源与便携式质谱仪联用快速检测平台,优化了喷雾电压和质谱入口毛细管温度,开发了高效快速的前处理技术。基于该平台和前处理技术,5种常规毒品(甲基苯丙胺、氯胺酮、可卡因、*O*^6^-单乙酰吗啡和3,4-亚甲双氧甲基苯丙胺)的尿液加标溶液的检出限为0.5~30 ng/mL,且其中4种毒品定量检测的线性相关系数大于0.99。除此之外,5种常规毒品在3个不同水平下的加标回收率为56.1%~103.7%,多次检测结果的相对标准偏差为9.0%~27.8%,说明联用检测平台与前处理方法结合可以达到良好的准确度。为了进一步检验该联用仪器的实战能力,测试了某社区戒毒康复中心40份阳性和110份阴性实际尿液样本,总体检测的准确率接近99%,且通过一次进样在20 s内可同时检测多种毒品。该研究成果有利于推动快速检测技术的发展,促进敞开式直接电离质谱仪技术的推广应用,提升一线执法服务水平。

## Abstract (en)

According to the Report of Drug Situation in China (2020), the growth rate of the number of drug abusers in China has decreased, but the number of drug abusers is still large. An efficient screening method is necessary for controlling drug abuse. As an important type of biological sample, urine is widely used for the rapid screening of drug addicts. However, because of the complex composition, low content, and strong interference from the body’s metabolism, the detection of drugs in urine remains a challenge. Traditional rapid screening techniques such as immunocolloidal gold analysis have a high false positive rate and insufficient quantitative capability. In addition, laboratory mass spectrometry methods require complicated time-consuming sample pre-processing and strict environmental conditions, and hence, are unsuitable for on-site rapid analysis. In recent years, various direct ionization mass spectrometry techniques such as direct analysis in real time (DART), desorption electrospray ionization (DESI), and dielectric barrier discharge ionization (DBDI) have advanced rapidly. These techniques have been applied to public safety, food safety, environmental detection, etc. In contrast to traditional ionization mass spectrometry methods, these direct ionization techniques allow for the *in situ* analysis of samples with simple or no pretreatment; moreover, they have the advantages of high analytical efficiency and sensitivity. In particular, pulsed electrospray ionization has the characteristics of less sample demand, compact, lightweight equipment, and no carrier gas. This paper presents a rapid method based on pulsed electrospray ionization mass spectrometry for the detection of urine samples. A rapid detection platform comprising a probe electrospray ionization source, a portable linear ion trap mass spectrometer (MS), and their coupling interface is adopted. The probe electrospray ion source includes a conducting metal wire, plastic handle, and silica glass capillary, whose tip has an inner diameter of 50 μm. The guide rail at the coupling interface is used to align the probe with the sample inlet of the portable mass spectrometer and maintain a distance of 10 mm between the probe tip and the sample inlet of the MS. The spray voltage of the probe electrospray ion source and the temperature of the MS inlet capillary are optimized at 1.8 kV and 205 ℃, respectively. In addition, rapid and efficient pretreatment techniques for urine samples have been developed. Buffer salts used for pH regulation and liquid-liquid extraction based on ethyl acetate were adopted for the pretreatment process. The linearity of the detection ability and the linear ranges of various drug-spiked solutions were also investigated. The results showed that the correlation coefficients for the quantitative detection of methamphetamine, ketamine, methylenedioxymethamphetamine (MDMA), and cocaine were greater than 0.99 at concentrations ranging from 1 to 100 ng/mL. Moreover, the limits of detection (LODs) for the five conventional drug-spiked urine were 0.5-30 ng/mL. The spiked recoveries ranged from 56.1% to 103.7%, with relative standard deviations (RSDs) of 9.0%-27.8%, implying that the combination of the instruments and the pretreatment method can lead to good accuracy. To validate the performance of the rapid detection method, 40 positive and 110 negative urine samples were tested and analyzed. The overall accuracy was over 99%, and the five conventional drugs in urine samples could be detected within 20 s. The research findings of this work could promote the development of rapid detection technology, accelerate the popularization and application of ambient direct ionization mass spectrometry, and improve the services of on-site law enforcement.

《2020年中国毒情形势报告》相关数据显示,目前国内毒品滥用人数增速减缓但吸毒群体规模依然较大。吸毒人员筛查技术是毒品监管和禁毒工作的重要支撑,为管控吸毒人员提供科学依据。自2011年《吸毒成瘾认定办法》实施以来,尿液中的毒品检测是常用的吸毒人员现场快速筛查手段之一,其检测结果也被认为是吸毒成瘾认定的标准。相比于体外毒品,尿液中的毒品具有成分复杂、含量低、机体新陈代谢干扰大等特点^[1]^,对高效的现场毒品分析技术提出了挑战。传统的免疫胶体金分析法存在假阳性和假阴性比例高、单次可检测物质种类少等缺点^[2]^;光谱法技术在检测混合物样品时,存在定量能力不足等问题^[3]^。实验室质谱技术是目前常见生物样本中毒品物质分析与检测手段的金标准方法,然而其对于单个样品检测前处理过程相对复杂^[4]^,使用环境要求相对苛刻,耗时较长,不满足一线的毒品现场快速检测需求。

近年来,直接电离质谱技术突飞猛进,如实时直接分析(DART)^[5]^、解吸电喷雾离子源(DESI)^[6]^、介质阻挡放电(DBDI)^[7,8]^等,不需要样品前处理或仅需简单的样品前处理即可实现样品原位分析,而且分析效率高,灵敏度高,已成功应用于公共安全、食品安全、环境检测等领域^[9,10]^。上述直接电离技术因需载气工作,导致进样装置相对复杂,从而限制了其在现场、便携式方向的应用。脉冲式电喷雾离子化技术^[11]^,具有样品需求量少、体积小、重量轻、无须载气等特点,已成功应用于实际样品中磺胺类物质的筛查和分析^[12]^;宁波华仪宁创智能科技公司在脉冲式电喷雾离子化技术的基础上研制了探针式电喷雾离子源,并已成功应用于毛发、污水等毒品检测仪器中。

本工作基于探针式电喷雾离子源与便携式质谱仪联用快检平台,建立面向生物样本的敞开式直接电离质谱技术的检测方法,旨在满足一线毒品检测的检测速度快、样品种类多、准确率高以及检出限低等需求。

## 1 实验部分

### 1.1 仪器与试剂

CRAIV-110便携式质谱仪、探针式离子源(宁波华仪宁创智能科技有限公司),探针喷头(武汉微探生物科技有限公司); Vortex-5涡旋仪(海门市其林贝尔仪器制造有限公司); TG16-WS台式高速离心机(湖南湘仪离心机仪器有限公司)。

纯净水(娃哈哈,杭州娃哈哈集团有限公司);乙腈和乙酸乙酯(色谱纯,美国TEDIA);磷酸氢二钠、氢氧化钠(国药试剂);甲基苯丙胺(methamphetamine)、氯胺酮(ketamine)、可卡因(cocaine)、*O*^6^-单乙酰吗啡(6-monoacetylmorphine)和3,4-亚甲双氧甲基苯丙胺(MDMA)5种标准品溶液(质量浓度100 μg/mL,公安部门)。

空白尿液样本的采集对象为云南省某市的5名健康男性,年龄区间在20~35岁。

### 1.2 实验方法

#### 1.2.1 样品制备

使用乙腈溶液分别对100 μg/mL的5种毒品标准液进行稀释,获得50 ng/mL的5种单标溶液。

分别取空白尿液300 μL,随后加入精确量取的*n* μL 100 μg/mL5种毒品标准液至1.5 mL离心管中,加入(1000-*n*) μL乙腈,涡旋1 min后配制1、4、8、10、20、40、50、100、200、300、1000 ng/mL等5种毒品的稀释溶液,待测。

将50 mL 0.05 mol/L磷酸氢二钠溶液与26.9 mL 0.1 mol/L氢氧化钠溶液混合均匀,加纯水稀释至100 mL,配制成pH 12的缓冲盐溶液。

#### 1.2.2 样品前处理

取300 μL待测尿液样品,加入300 μL pH 12的缓冲盐溶液,静置1 min,加入300 μL乙酸乙酯直接萃取,静置1 min,之后涡旋30 s,离心1 min,最后用探针蘸取上层有机相约1 μL待测。

### 1.3 仪器条件

便携式质谱仪见图1-Ⅰ。采样毛细管:内径为0.37 mm,长度为100 mm,直流电压为90 V;线性离子阱:共振激发所用交流电的电压为1~4 V,射频(radio frequency, RF)幅值电压为120~1500 V, RF频率为1.08 MHz;打拿极电压:-5500 V;倍增器电压:-1100 V;选择标准模式进行质量扫描,扫描范围为*m/z* 50~700,仪器分辨率为1 Da;前级真空压力:0.69 kPa。

**图 1:**
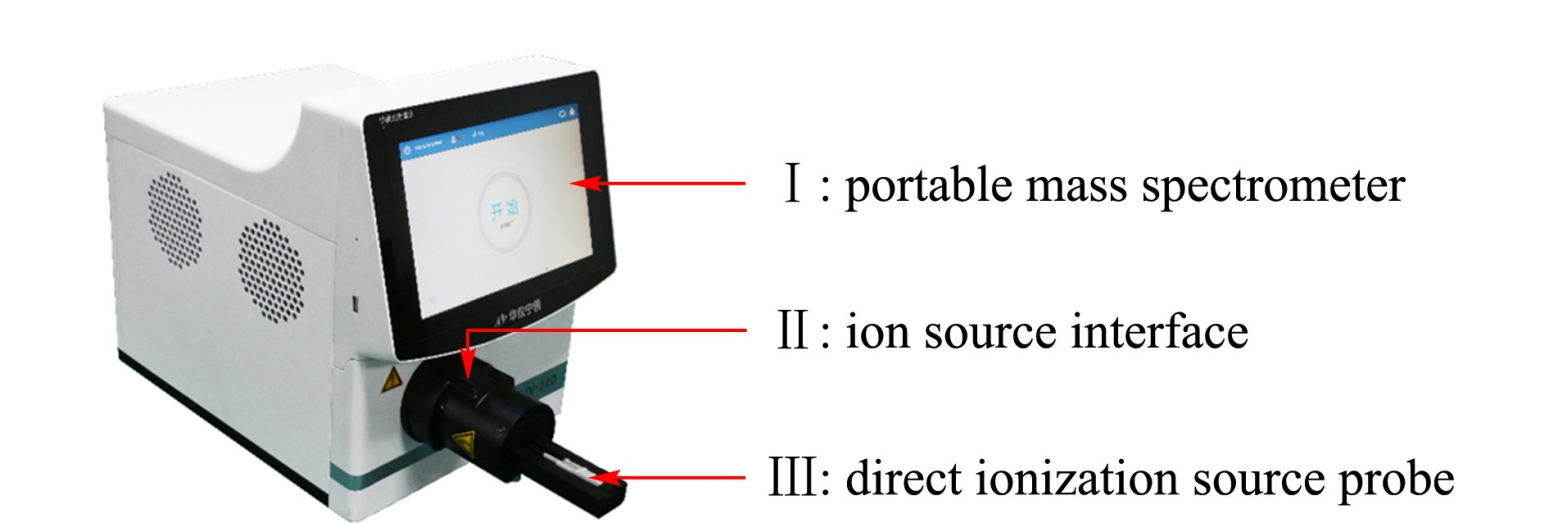
探针式电喷雾离子源-便携式质谱仪联用系统

探针式离子源见图1-Ⅲ。离子源极性:正离子模式(ESI^+^);探针喷头为石英玻璃毛细管:外径为1.5 mm,内径为1.1 mm,长度为5 cm;喷头针尖口径:50 μm;喷头针尖到质谱仪入口距离:10 mm。

MS^2^分析质谱参数见表1。

**表 1:** 各毒品的MS^2^分析参数

Drug	Parent ion (m/z)	Daughter ions (m/z)	Isolation voltage/V	q	Fragmentation voltage/V	Ion injection time/ms
Methamphetamine	150	91^*^, 119	2.5	0.40	2	50
Methylenedioxymethamphetamine (MDMA)	194	163^*^, 135, 105	3.0	0.40	3	50
Ketamine	238	220^*^, 179, 125	3.0	0.35	3	50
Cocaine	304	182^*^, 150	3.0	0.35	3	50
6-Monoacetylmorphine	328	211^*^, 165	4.0	0.40	4	50

* Quantitative ion; *q*: trapping parameter of ion trap.

## 2 结果与讨论

### 2.1 仪器条件的优化

#### 2.1.1 喷雾电压

为了使探针式电喷雾离子源-便携式质谱仪达到最佳的检测状态,有必要提高离子化效率和提升离子的传输效率,因此需对探针式离子源的喷雾电压和便携式质谱仪的毛细管温度进行优化。由于敞开式离子源信号难免受到各种因素干扰,导致质谱信号波动,因此本工作采用计算平均峰强的方法来减小信号波动所带来的误差,离子强度信号的波动可用相对标准偏差(RSD)来表征。

使用50 ng/mL的可卡因乙腈溶液作为测试样品,待检测前用等体积乙酸乙酯进行萃取,蘸取上层有机相直接分析,并以二级质谱分析模式下可卡因的子离子(*m/z* 182)强度作为考察指标,其中每个数据点采样5次,且每次更换不同探针。该强度取值方式为:在整个采样时间内,当5 s后某一时刻的离子强度下降到最初5 s时间内离子强度平均值的1/2时,则将该段时间内的离子强度的平均值作为有效强度。在0.3~4.8 kV喷雾电压范围内,考察了探针式电喷雾离子源的喷雾电压对离子信号强度和喷雾时间的影响。由图2a可以看出,随着喷雾电压从0.3 kV升高至1.3 kV,信号强度从零开始陡升,这是因为施加的喷雾电压在1.3 kV时,达到液体表面产生电荷分离所需的电场的阈值电压^[13]^。在1.3~3.3 kV时随着喷雾电压的增加,信号强度出现小幅度的先下降后上升,这是因为随着电压的不断增大喷雾频率开始加大,产生的喷雾液滴出现不稳定状态^[14]^;在电压超过3.3 kV之后,喷雾频率增加且喷雾液滴变大,去溶剂化效果变差^[15,16]^,从而导致离子在质谱仪内传输时损失增加。相比在1.3~3.3 kV喷雾电压下的各信号响应强度,喷雾电压为1.8 kV时,信号强度波动较小。

**图 2:**
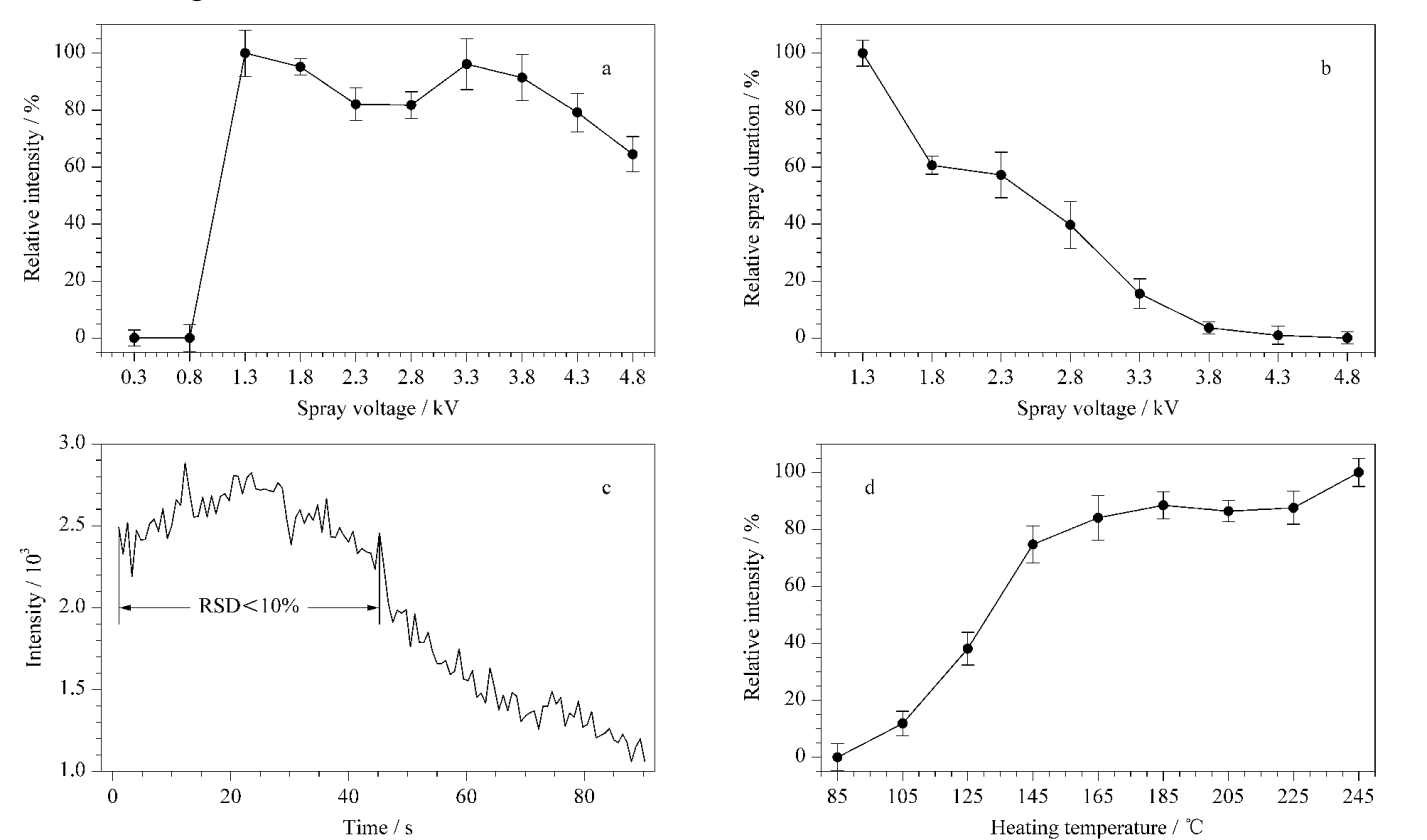
(a)相对离子强度和(b)相对喷雾时间与喷雾电压关系,(c)喷雾电压为1.8 kV时离子强度随时间变化曲线, (d)相对离子强度与传输毛细管温度的关系(*n*=5)

如图2b所示,随着喷雾电压的不断增大,产生喷雾的持续时间(计算离子有效强度时其对应的时长)与其呈负相关,因为在探针内溶液量一定的情况下,喷雾电压越大喷雾的速度就越快,喷雾持续时间就越短。当喷雾电压在1.3 kV以下时,无法产生喷雾,因此图2b不包含该范围内数据。

当喷雾电压设为1.8 kV时,离子峰强度随时间变化曲线如图2c所示,可以看到离子峰强度在前45 s内相对比较稳定,RSD<10%,随后信号强度逐渐下降。考虑到信号稳定性和喷雾时长对数据分析的影响,除特殊说明外,本工作后续所有实验的喷雾电压均设置为1.8 kV,所有离子峰强度检测均采用喷雾前20 s内的有效强度数据计算平均值。

#### 2.1.2 进样口温度

由于便携式质谱仪的传输毛细管温度会对离子源喷雾液滴的脱溶剂化及毛细管内带动离子传输的气流状态产生影响^[17]^,因此考察了不同毛细管温度对信号强度的影响。同样以50 ng/mL的可卡因溶液做测试,其子离子(*m/z* 182)强度作为考察目标,其中每个数据点采样5次,且每次更换不同探针,其离子信号响应强度与温度的关系如图2d所示。当质谱毛细管温度在85~145 ℃范围内时,信号强度随着温度的提高而增大,这是因为随着温度的升高传输毛细管内的气流从湍流变成了层流^[18]^,离子因撞击到毛细管内壁上而造成的损失大大降低。当温度在145~245 ℃范围内,信号强度的曲线波动较小且呈现出缓慢上升的趋势,说明提高温度有利于提升脱溶剂化效果,离子在质谱内的传输效率得到提高。这是因为信号强度的提升超过了因温度升高扩散作用增强而带来的损失。当温度为185~205 ℃时,信号强度相对稳定且差异性不大,其中温度为205 ℃时,RSD相对较小;综合权衡了质谱毛细管加热装置的寿命和密封圈的耐热性后,本工作后续实验将质谱毛细管温度设为205 ℃。

综上,将探针式电喷雾离子源的喷雾电压设置为1.8 kV,便携式质谱仪的毛细管温度设置为205 ℃。

### 2.2 标准溶液检测

使用50 ng/mL的甲基苯丙胺、氯胺酮、可卡因、*O*^6^-单乙酰吗啡和MDMA 5种单标溶液,在检测前用等体积乙酸乙酯进行萃取,使用探针蘸取上层有机相直接分析,采用正离子质谱模式,并同时记录MS^1^和MS^2^质谱数据,结果如图3所示。可以看到探针式电喷雾离子源结合便携式质谱仪不仅能够准确定性目标样品,而且检测出的二级子离子也具有较高的信噪比。

**图 3:**
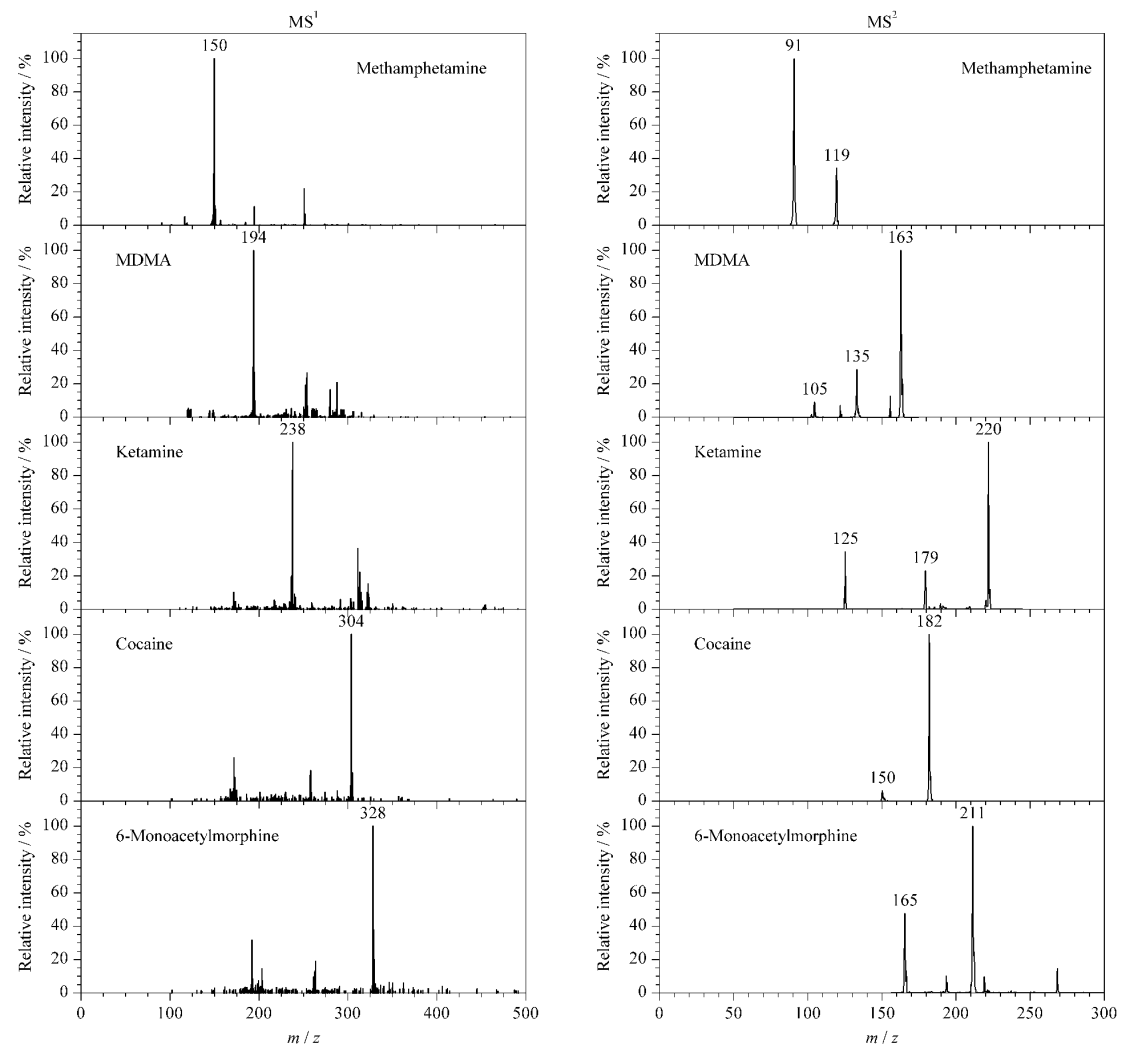
5种常见毒品标准溶液的MS^1^和MS^2^谱图

### 2.3 线性范围、检出限和回收率

在前述仪器优化后的条件下,通过对1.2.1节中获得的1、4、8、10、20、40、50、100 ng/mL的5种毒品稀释溶液采用1.2.2节方法进行前处理,每种毒品的每个加标溶液进行5次检测且每次更换不同探针,其在各自的线性范围内响应关系如表2所示。从表2中可以看到,尿液基质加标的5种毒品质谱信号呈现良好的线性关系,甲基苯丙胺、氯胺酮、MDMA、可卡因毒品的子离子信号强度和质量浓度之间的相关系数(*R*^2^)均大于0.99,线性关系良好,说明该尿液前处理方法与联用检测平台结合使用具有优良的检出能力。

**表 2:** 尿液基质中5种常见毒品的线性范围、相关系数和相对标准偏差(*n*=5)

Drug	Regression equation	R^2^	Linear range/(ng/mL)	RSDs/%
Methamphetamine	y=0.1621x+0.6399	0.9983	4-50	6.9-17.8
MDMA	y=1.5734x-5.5052	0.9969	10-100	10.7-27.9
Ketamine	y=0.3811x+0.9830	0.9983	10-100	8.8-22.5
Cocaine	y=0.4079x+1.1997	0.9929	10-100	10.6-31.4
6-Monoacetylmorphine	y=0.0317x+0.4315	0.9891	20-100	28.3-45.7

*y*: ion intensity; *x*: mass concentration, ng/mL.

为了验证1.2.2节前处理方法的有效性,使用探针式电喷雾离子源联用便携式质谱仪检测平台分别对空白尿液样本进行了常规5种毒品不同水平的加标回收测试。空白尿液中各物质的加标水平分别为10、50、100 ng/mL,各加标样品均进行5次平行实验,采用定量子离子强度计算回收率和RSD,结果见表3,可以看出在3个水平下的加标回收率为56.1%~103.7%,相对标准偏差为9.0%~27.8%。

**表 3:** 5种毒品在尿液中的加标回收率、相对标准偏差和检出限

Compound	Spiked at 10 ng/mL			Spiked at 50 ng/mL			Spiked at 100 ng/mL		LOD/ (ng/mL)
	Recovery/%	RSD/%		Recovery/%	RSD/%		Recovery/%	RSD/%	
Methamphetamine	56.1	27.8		77.4	17.3		94.4	9.0	0.5
MDMA	62.4	24.3		91.0	12.6		103.7	10.3	10
Ketamine	73.7	27.2		84.5	17.8		95.5	8.9	10
Cocaine	67.2	27.0		85.2	14.9		92.1	10.6	10
6-Monoacetylmorphine	-	-		60.7	24.6		81.4	15.8	30

-: Data are not available.

以信噪比(*S/N*)大于或等于3作为判断是否检出的依据,5种毒品的检出限(LOD)为0.5~30 ng/mL,这表明仪器系统具有较高的灵敏度和准确性,对尿液中毒品检测具有较好的适用性。

### 2.4 实际尿液样本测试

针对云南某地社区戒毒康复中心提供的一批实际尿液样本(已进行过司法鉴定标准检测),利用本工作建立的方法进行复检,进一步验证其在实际样品检测中的适用性。检测样本中同时吸食甲基苯丙胺(俗称冰毒)与吗啡的样本数为5例,吸食冰毒的样本数为10例,吸食吗啡的样本数为25例,阴性样本数为110例。

采用联用检测平台完成所有样本的检测耗时为55 min,无论对单个样本中的一种毒品还是多种毒品的检测耗时均不超过20 s。检测结果表明,5例多组分混检(multi-component detection)的样本(其中含有冰毒和吗啡两种毒品)均能够在所开发的尿液前处理方法下使用联用检测平台准确检出,仅1例吗啡的阳性样本未检出,其他样本检测结果均与司法鉴定标准检测结果一致,整体的检测准确率超过99%。吗啡样本检测结果之所以存在未检出的现象,这可能是因为该物质在人体内与葡萄糖醛酸容易结合形成吗啡-3-葡萄糖醛酸苷等其他物质^[19]^,影响到该目标物的检测。

总体而言,利用联用检测平台结合所建立的尿液前处理方法,不仅检测所需时间短而且能对样本目标物进行混检,能够适用于实际尿液样本的检测并具有较高的准确率,其对甲基苯丙胺的检出限接近司法鉴定检测标准^[20]^,且所有常规毒品检出限都远低于胶体金行业标准(GA/T 1666-2019, YY/T 1595-2017, GA/T 1668-2019, GA/T 1692-2020)。

## 3 结论

本工作基于探针式电喷雾离子源与便携式质谱仪联用检测平台,考察了探针式电喷雾离子源的喷雾电压与质谱仪毛细管入口温度对仪器性能的影响,开发了一套用于尿液生物检材的前处理技术,验证了该联用检测平台结合前处理方法的检测灵敏度、适用性、定性能力以及对实际样本的检测水平和准确性。结果表明该方法具有检出限低、操作简便、快速、准确等优点,解决了传统快筛技术存在假阳率高、定量能力不足的问题,并针对实验室质谱技术在快速检测中存在的前处理复杂、检测耗时长、使用环境苛刻等问题给出了有效替代方案,可进一步拓宽敞开式直接电离质谱技术在现场快速检测领域中的应用,并可作为现场吸毒人员快速筛查的有力“武器”,提升一线实战水平,为健康中国、平安中国建设做出贡献。
